# Dynamic Contrast-Enhanced CT Characterization of Xp11.2 Translocation/TFE3 Gene Fusions versus Papillary Renal Cell Carcinomas

**DOI:** 10.1155/2015/298679

**Published:** 2015-11-09

**Authors:** Jian He, Kefeng Zhou, Bin Zhu, Gutian Zhang, Xiaogong Li, Hongqian Guo, Weidong Gan, Zhengyang Zhou, Tian Liu

**Affiliations:** ^1^Department of Radiology, Nanjing Drum Tower Hospital, The Affiliated Hospital of Nanjing University Medical School, Nanjing 210008, China; ^2^Department of Urology, Nanjing Drum Tower Hospital, The Affiliated Hospital of Nanjing University Medical School, Nanjing 210008, China; ^3^Radiation Oncology and Winship Cancer Institute, Emory University, Atlanta, GA 30322, USA

## Abstract

*Purpose*. To compare the differences of CT characteristics between renal cell carcinomas (RCCs) associated with Xp11.2 translocation/TFE3 gene fusions (Xp11.2 RCCs) and papillary cell renal cell carcinomas (PRCCs).* Methods*. CT images and clinical records of 64 patients (25 Xp11.2 RCCs, 15 type 1 and 24 type 2 PRCCs) were analyzed and compared retrospectively.* Results*. Xp11.2 RCC more frequently affected young (30.7 ± 8.7 years) women (16/25, 64%) with gross hematuria (12/25, 48%), while PRCC more frequently involved middle-aged (54.8 ± 11.1 years) men (28/39, 71.8%) asymptomatically. Xp11.2 RCC tended to be heterogeneous density with some showing circular calcification. Lesion sizes of Xp11.2 RCC (5.4 ± 2.2 cm) and type 2 PRCC (5.7 ± 2.5 cm) were significantly larger than that of type 1 PRCC (3.8 ± 1.8 cm). Xp11.2 RCC contained more cystic components (22/25, 88%) than type 1 PRCC (all solid) and type 2 PRCC (9/24, 36.0%). Type 1 PRCC (13/15, 86.7%) and Xp11.2 RCC (21/25, 84.0%) showed more clear boundary than type 2 PRCC (12/24, 50.0%).* Conclusion*. CT features including diameter, boundary, attenuation, nature, and circular calcification of the tumor, combined with demographic information and symptoms, may be useful to differentiate Xp11.2 RCC from different subtypes of PRCC.

## 1. Introduction

Renal cell carcinoma (RCC) associated with Xp11.2 translocation/TFE3 gene fusions (Xp11.2 RCC) was introduced as a genetically distinct entity into the World Health Organization classification of renal neoplasms in 2004 [1]. Microscopically, Xp11.2 RCC shows various features, including abundant clear or eosinophilic cytoplasm, irregular nuclei with vesicular chromatin, and prominent nucleoli with papillary, nested, alveolar, or tubular architectures. Although Xp11.2 RCC is predominantly diagnosed in children and rare in adults, the disease seems more advanced and aggressive in adults than in children [2]. Moreover, based on meta-analysis, Xp11.2 RCC has a poorer prognosis than non-Xp11.2 RCC in children and young adults [3].

Previous studies with computed tomography (CT) have shown that Xp11.2 RCC appears as a large, well-defined cystic-solid renal mass with intratumoral hemorrhage and circular calcification. These features are especially evident in young females with hematuria [4–9]. In our previous study, dynamic contrast-enhanced CT (DCE-CT) showed heterogeneously moderate prolonged enhancement of Xp11.2 RCC. Different from Xp11.2 RCC, DCE-CT of clear cell RCC (CCRCC) had a typical “wash-in and wash-out” pattern, microscopically showing nests of epithelial cells with clear cytoplasm and a distinct cell membrane, separated by a delicate branching network of vascular tissue [10].

Papillary RCC (PRCC), the second most common RCC subtype, is histologically characterized by a predominantly papillary growth pattern composed of columnar/cuboidal cells and contains two histological types with distinct behavior and prognosis [11]. Type 1 PRCC contains small cells with scanty pale cytoplasm and small ovoid nuclei that are arranged in a single layer on the basement membrane of the papillary core. Type 2 PRCC contains cells with abundant eosinophilic cytoplasm, large and spherical nuclei, prominent nucleoli, and varying degrees of nuclear pseudostratification.

There are significant challenges to distinguish Xp11.2 RCC from PRCC. Considerable similarities exist in the microscopic morphologies of Xp11.2 RCC and PRCC, causing frequent, pathology misdiagnosis [8]. It is also hard to differentiate between Xp11.2 RCC and PRCC with CT or magnetic resonance imaging (MRI) because both are hypovascular neoplasms [12, 13]. Nevertheless, identifying the correct RCC subtype is important, as Xp11.2 RCC and PRCC have different behaviors and prognosis. PRCC is usually described as a single entity that has a favorable outcome compared with CCRCC, while Xp11.2 RCC exhibits higher invasiveness and poorer prognosis than CCRCC [3]. Radical operation has served as the main treatment regimen for patients with Xp11.2 RCC, whereas patients with PRCC can be treated with partial nephrectomies.

Current radiologic literature lacks comparative studies that distinguish the RCC subtypes. For example, most radiological studies evaluated PRCC as a single subtype [12, 14, 15], and only a few studies focused on the differential diagnosis between the two histological subtypes [16]. Moreover, the difference between Xp11.2 RCC and subtypes of PRCC on DCE-CT has never been reported.

The aim of this study was to compare the difference between Xp11.2 RCC and PRCC (including type 1 and type 2) on DCE-CT.

Awareness of imaging differences between various subtypes of RCC may help promote further confirmatory diagnostic processes including immunohistochemical (IHC) staining and fluorescence in situ hybridization (FISH) assay and may help improve treatment strategy [4].

## 2. Materials and Methods

### 2.1. Ethics Statement

This retrospective study was approved by the institutional review board. The informed consent was waived due to the retrospective nature of this study.

### 2.2. Patients

From January 2007 to January 2015, a total of 25 consecutive adult patients (≥18 years old) with Xp11.2 RCC who had undergone a radical or partial nephrectomy in our institution were retrospectively identified. From 113 patients diagnosed with PRCC, based on microscopic findings, a subset of 39 PRCC-patients with a definite negative FISH assay result were selected for this comparative study. The other 74 patients failed to undergo FISH analysis due to lack of tissue specimens and as a result, therefore, were excluded from this study because a diagnosis of Xp11.2 RCC cannot be excluded solely based on microscopic findings. None of the enrolled patients had received local or systematic therapy before CT scanning and surgery.

### 2.3. Clinical, Treatment, and Pathological Information

A total of 25 Xp11.2 RCCs and 39 PRCCs were included in this study. Each patient had one lesion. Six Xp11.2 RCC patients were diagnosed at stage 3 and stage 4, while the majority of PRCC patients (34/39) were diagnosed at stage 1 and stage 2. Clinical, radiologic, and pathological records of the 2 RCC subtypes are shown in Table 1. No history of malignancy, chemotherapy, or toxic exposure was recorded in the enrolled patients.

Open or laparoscopic radical operation served as the main treatment for 18 patients with Xp11.2 RCC, and the remaining 7 patients underwent laparoscopic partial nephrectomy, while 23 out of 39 patients with PRCC underwent laparoscopic or open partial nephrectomies, 15 patients received radical nephrectomies, and the remaining 1 patient underwent radiofrequency ablation. Although none of the patients in both groups died during surgery, 3 patients with Xp11.2 RCC and one patient with type 2 PRCC died of distant metastasis during a follow-up between 6~78 months (mean, 36.6 months; medium, 33 months).

Gross pathological record of the specimen was reviewed by 2 pathologists to confirm the tumor's location, boundary, capsule, shape, necrotic and cystic components, hemorrhage, and tumor thrombosis observed on CT imaging. IHC staining with TFE3 antibody and FISH assay with a self-designed polyclonal break-apart probe confirmed the diagnosis of Xp11.2 RCC in 25 cases. However, both TFE3 staining and FISH assay were negative in all PRCC cases.

### 2.4. CT Examination

All patients underwent unenhanced and DCE-CT scans using a multidetector CT scanner (LightSpeed; GE Healthcare, Princeton, NJ) with a 5.0 mm slice thickness at 40, 70~80, and 180 seconds to obtain corticomedullary, nephrographic, and delayed phases, after injection of 1.2 mL/kg body weight of contrast media (Omnipaque 350 mg I/mL; GE Healthcare, US), at a rate of 3.0 mL/s followed by 40 mL saline solution using a power injector (Medrad Stellant, Indianola, PA). Images were obtained at a tube voltage of 120 kVp, a tube current of 240 mA, with a rotation time of 0.6 seconds, a helical pitch of 1.375, a field view of 35 to 40 cm, and a matrix of 512 × 512.

### 2.5. Image Interpretation

All CT images were reviewed in consensus by 2 radiologists (Jian He and Kefeng Zhou with 5- and 10-year experience in abdominal CT diagnosis, resp.). The images were reviewed on a picture archiving and communication system workstation (GE AW4.3 workstation).

Tumor features on CT imaging were evaluated based on the following criteria:

- Tumor location: the tumor was located in the left or right kidney, with cortical, cortical-medullary, or medullary involvement.
- Tumor size: the maximum diameter of the tumor was measured in centimeters.
- Tumor boundary: a clear boundary was characterized by well-defined, bulging tumor margins that displaced surrounding structures. An unclear boundary was defined as lacking clear borders between the tumor and surrounding structures.
- Tumor shape: a regular shape was characterized as round or oval. Irregular shapes included a roughly round or oval tumor with focal protrusions and lobulated and infiltrative grow patterns.
- Tumor texture: a solid tumor had soft tissue density without obvious necrotic or cystic areas. A cystic-solid tumor had solid and cystic components. A cystic tumor was completely cystic with a capsule wall. Necrotic or cystic components were defined as the irregular unenhanced cavitation on contrast-enhanced CT images.
- Presence of intratumoral hemorrhage: intratumoral hemorrhage presented as patchy or formless hyperdense area on unenhanced CT scan (CT value 40~70 Hounsfield Unit, HU), nonenhancing on enhanced CT scan.
- Presence of intratumoral calcification: calcification presented as dense foci (>100 HU). Number, shape, and distribution of calcification were recorded.
- Presence of intratumoral fat: fat showed a hypodense area (−50 to −100 HU) on unenhanced CT scan.
- Presence of tumor thrombosis: the tumor was found in the lumen of the renal vein or the inferior vena cava.
- Presence of local lymphadenopathy: retroperitoneal nodal was enlarged with a short-axis diameter at least 10 mm.
- Tumor metastasis: presence of distant metastasis in other organs, such as the liver and lung nodules, which were enlarged during follow-up.
- Tumor attenuation (HU) in unenhanced, corticomedullary, nephrographic, and delayed phases: computed tomographic attenuation values (in HU) of the tumor were measured on each phase by the 2 radiologists. The region of interest (ROI) was defined in the solid portion of the mass to avoid intratumoral calcification and cystic and necrotic components in the slice with maximum diameter of the lesion. For all images, each 100 mm^2^ ROI was measured 3 times by both radiologists, and the mean value was used.

### 2.6. Statistical Analysis

Statistical analysis was performed using SPSS 13.0 software (SPSS Inc., Chicago, IL). Numeric data were expressed as mean ± standard deviation, and categorical data were expressed as percentages. Evaluated characteristics were compared between the RCC subtypes using the repeated measures analysis of variance (ANOVA) or *χ* ^2^ test. A *P* value less than 0.05 was considered statistically significant.

## 3. Results

### 3.1. Xp11.2 RCC and PRCC

**Figure 1:**
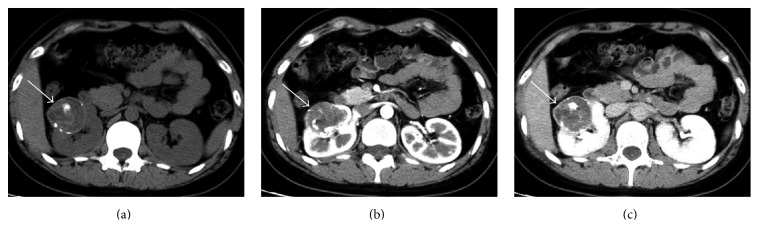
Renal cell carcinoma associated with Xp11.2 translocation/TFE3 gene fusions in a 22-year-old woman with gross hematuria. (a) Abdominal plain CT scan shows a round well-defined mass of 5.0 cm in size with mixed intensity (around 50 HU) in the right kidney. Note the punctate and circular calcification within and around the lesion. (b) The tumor, which involves both the cortex and medulla, shows heterogeneously moderate enhancement (to around 84 HU) during corticomedullary phase. (c) The tumor continues to be enhanced heterogeneously from corticomedullary phase to nephrographic phase (to around 90 HU). The unenhanced areas within the tumor indicate necrosis.

The clinical, pathological details, and tumor characteristics on CT in Xp11.2 RCC and PRCC are shown in Table 1. Xp11.2 RCC more frequently affected young (30.7 ± 8.7 years) women (16/25, 64%) with gross hematuria (12/25, 48%), while PRCC more frequently involved middle-aged (54.8 ± 11.1 years) men (28/39, 71.8%) without obvious symptoms. Xp11.2 RCC was more heterogeneous (20/25, 80%) and with cysts (22/25, 88%). Circular calcification was observed in Xp11.2 RCC (15/25, 60%) more often than PRCC (6/39, 15.4%). A case of Xp11.2 RCC is shown in Figure 1.

### 3.2. Type 1 and 2 PRCCs

Type 2 PRCC was significantly larger (5.8 ± 2.5 cm) than type 1 PRCC (3.6 ± 1.6 cm) (*P* = 0.002). Type 2 PRCC was more likely to be heterogeneous (16/24, 66.7%) than type 1 PRCC (3/15, 20%). All type 1 PRCCs were solid, while 62.5% (15/24) of type 2 PRCC was solid. Type 1 PRCC had more clear boundaries (13/15, 86.7%) than type 2 PRCC (12/24, 50%) (*P* = 0.020).

### 3.3. Xp11.2 RCC and Type 1 and 2 PRCCs

**Figure 2:**
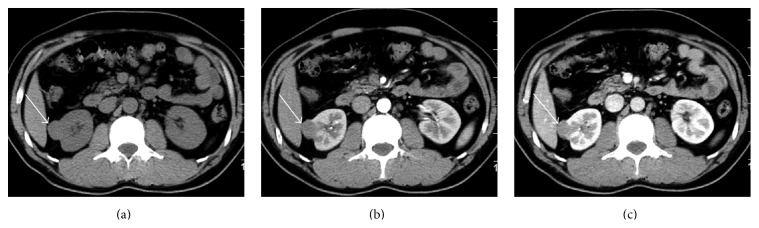
Type 1 papillary renal cell carcinoma in a 39-year-old asymptomatic man. (a) Abdominal plain CT scan shows a round well-defined isodense (40 HU) nodule of 2.5 cm in size in the cortex of right kidney. (b) The tumor is enhanced slightly and homogeneously (to around 58 HU) during corticomedullary phase without necrotic or cystic areas in the lesion. (c) The tumor continues to be enhanced homogeneously (to around 78 HU) during the nephrographic phase.

**Figure 3:**
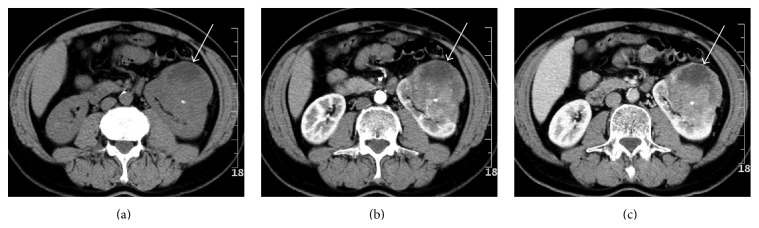
Type 2 papillary renal cell carcinoma in a 58-year-old asymptomatic man. (a) Abdominal plain CT scan shows an irregular ill-defined heterogeneous (around 44 HU) mass of 8.3 cm in size in the left kidney. Note a punctate calcification within the lesion. (b) The tumor, which involves both the cortex and medulla, shows heterogeneously moderate enhancement (to around 80 HU) during corticomedullary phase. (c) The tumor's attenuation remains around 80 HU in the nephrographic phase. The unenhanced areas within the tumor indicate necrosis.

Xp11.2 RCC was significantly larger (5.4 ± 2.2 cm) than type 1 PRCC (3.6 ± 1.6 cm) (*P* = 0.009). Xp11.2 RCC (20/25, 80%) was more heterogeneous than type 1 PRCC (3/15, 20%) (*P* < 0.001). Xp11.2 RCC was often cystic-solid (22/25, 88%), while type 1 PRCC was all solid. Xp11.2 RCC was more likely to have clear boundaries (21/25, 84%) than type 2 PRCC (12/24, 50%) (*P* = 0.011). Xp11.2 RCC contained more cystic components (22/25, 88%) than type 2 PRCC (9/25, 36%) (*P* < 0.001). One case of type 1 PRCC and one case of type 2 PRCC are shown with Figures 2 and 3, respectively.

### 3.4. DCE-CT of Xp11.2 RCC and PRCC

**Figure 4:**
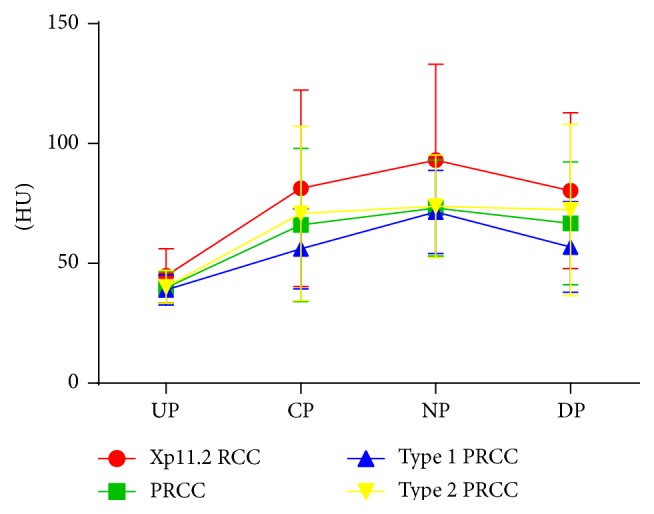
Dynamic contrast enhancement patterns of Xp11.2 RCC, type 1 and 2 PRCCs. Xp11.2 RCC: renal cell carcinoma associated with Xp11.2 translocation/TFE3 gene fusions; PRCC: papillary renal cell carcinoma; UP: unenhanced phase; CP: corticomedullary phase; NP: nephrographic phase; DP: delayed phase; HU: Hounsfield unit.

Both Xp11.2 RCC and PRCC showed moderately prolonged enhancement on DCE-CT and peaked at nephrographic phase (Figure 4). Tumor attenuations of Xp11.2 RCCs were significantly higher than those of PRCCs in plain and nephrographic phase scans (Table 2).

## 4. Discussion

### 4.1. Comparison between Xp11.2 RCC and PRCC

Xp11.2 RCC is characterized by various translocations involving chromosome X, all resulting in gene fusions involving the TFE3 gene [2]. Xp11.2 RCC usually affects children and young adults, with a slight female predominance (64.0% in our study) [9]. Most Xp11.2 RCCs (23/25, 92%) in this study involved both the cortex and medulla simultaneously, which were consistent with Wang et al.'s report [8] and probably responsible for the high incidence of hematuria (12/25, 48%). Most Xp11.2 RCCs (21/25; 84.0%) had clear boundaries, which were consistent with Zhu et al.'s report [6], probably due to fibrous capsules of the tumor. Based on previous studies, including our own, Xp11.2 RCC presented as a heterogeneous mass with necrotic or cystic components and intratumoral hemorrhage [4–10]. Calcification was commonly detected by CT in Xp11.2 RCC [5, 7–9], and pathological observation confirmed the formation of psammoma bodies in the tumor [2]. We reported that circular calcification around or within the tumor is a specific clue for CT diagnosis of Xp11.2 RCC [10]. On unenhanced CT scans, most Xp11.2 RCCs appear hyperdense relative to the renal parenchyma. After the injection of contrast media, Xp11.2 RCC showed heterogeneous moderately prolonged enhancement on DCE-CT [10].

PRCC also bears distinct molecular genetic and histologic characteristics [17]. Commonly seen among patients over 55 years [1], PRCC is usually a well-defined tumor containing a fibrous capsule [17]. On unenhanced CT scans, PRCC shows isoattenuation or hyperattenuation compared with that of normal renal parenchyma [18]. Typically hypovascular and homogeneous [19] PRCC shows lower enhancement than CCRCC and peaks in the nephrographic phase on dynamic CT studies [20].

Clinically speaking, Xp11.2 RCC often affects young women with gross hematuria, while PRCC is more likely to occur in old men without specific symptoms. In our series, majority of patients with Xp11.2 RCC (18/25) underwent radical nephrectomy, whereas 23 patients with PRCC (79.3%) underwent partial nephrectomies in this study.

Hence, preoperative CT differentiation between those two entities is of great importance for treatment planning. There were no significant differences in diameter and location (cortical/cortical-medullar/medullar) between Xp11.2 RCC and PRCC. They shared similar boundary and shape. Both of them contained hemorrhages without fat content. Xp11.2 RCC was more heterogeneous and contained more cystic components, while PRCC was more homogenous and presented as a solid entity. Circular calcification was more often observed in Xp11.2 RCC than in PRCC. Tumor attenuation of Xp11.2 RCC (44.8 ± 11.2 HU) was significantly higher than that of PRCC (39.8 ± 6.6 HU) in plain CT scans by the repeated measures of ANOVA (*P* = 0.032). Although both Xp11.2 RCC and PRCC showed moderate prolonged enhancement, the tumor attenuation of Xp11.2 RCC (93.1 ± 40.0 HU) was significantly higher than PRCC (73.1 ± 20.0 HU) (*P* = 0.021) in the nephrographic phase.

### 4.2. Comparison between Type 1 and 2 PRCCs

In radiology literatures, PRCC has usually been described as a single entity that has a favorable outcome [12, 14, 15]. However, in urologic studies, researchers suggested that PRCCs are a heterogeneous group of entities with different pathologic behaviors [11]. In this study all type 1 PRCCs were at stage I/II without any tumor thrombosis, lymph node, or distant metastasis, while 5 patients with type 2 PRCCs (20.8%) were at more advanced stages with more metastasis.

However, the imaging features of type 1 and 2 PRCCs were very similar. There were no significant differences of location, shape, hemorrhage, calcification, and fat between them. In this study, the mean attenuations of type 1 and 2 PRCCs were similar in unenhanced phase (38.9 ± 6.3 versus 40.2 ± 6.8 HU, *P* = 0.44), which was consistent with previous studies [16]. Both subtypes showed moderate prolonged enhancement on DCE-CT. Tumor attenuation of type 2 PRCC appeared slightly higher than that of type 1 in corticomedullary phase (70.8 ± 36.4 versus 56.0 ± 16.7 HU) without significant difference (*P* = 0.496) (Table 2), which suggested that neither enhancement pattern nor enhancement degree was helpful in discriminating them.

To our knowledge, there were no large studies that showed any specific features that can differentiate between type 1 and 2 PRCCs. We found that type 1 PRCC (3.6 ± 1.6 cm) was significantly smaller than type 2 PRCC (5.8 ± 2.5 cm) (*P* = 0.002), which is contradictory with Mydlo et al.'s report [21], but consistent with most other studies [19, 22, 23]. It was reported that type 2 PRCC grew faster than type 1 [16]. The margin of type 1 PRCCs was more distinct than type 2 PRCC, which is consistent with previous reports [19, 22, 23]. Type 1 PRCC had more homogeneous density than type 2 PRCC. All type 1 PRCCs in this study were solid, while 37.5% (9/24) of type 2 PRCCs contained cystic components, which proved clues for differential diagnosis between those two distinct types.

### 4.3. Comparison between Xp11.2 RCC and Type 1 and 2 PRCCs

There were no significant differences of location, hemorrhage, and fat between Xp11.2 RCC and type 1 PRCC. Both of them showed clear boundary and regular shape. However, Xp11.2 RCC was significantly larger and more heterogeneous than type 1 PRCC. Many Xp11.2 RCCs contained cysts (22/25, 88%), while type 1 PRCCs were all solid lesions (12/12, 100%) without cystic degeneration or necrosis. In addition, some Xp11.2 RCCs contained circular calcification (10/25, 40%), which was seldom detected in type 1 PRCC (1/12, 8.3%). Xp11.2 RCC enhanced heterogeneously, while type 1 PRCC enhanced homogenously on DCE-CT.

Except for gender, age, and symptoms, Xp11.2 RCC and type 2 PRCC shared similar stage, behavior, and prognosis. Moreover, CT findings of Xp11.2 RCC and type 2 PRCC were also quite similar and difficult to differentiate between each other. Both of them were relatively large, involving both cortex and medullar of the kidney. They had similar shape and attenuation and contained hemorrhage, circular calcification without fat content. Nevertheless, Xp11.2 RCC had clearer boundaries and more cystic components than those of type 2 PRCC.

### 4.4. Differential Diagnosis between Other Subtypes of RCCs

We have compared Xp11.2 RCC with CCRCC and found that contrast-enhanced pattern and degree differed significantly between these two entities [10]. A tumor-to-cortex ratio in corticomedullary phase <0.62 gave a sensitivity of 90.0% and a specificity of 92.9% in differentiating Xp11.2 RCC from CCRCC (AUC = 0.957, *P* < 0.001) [10]. Zhu et al. compared the multislice CT findings of Xp11.2 RCC and collecting duct carcinoma and found that distinguishing features including density on unenhanced CT, enhancement patterns, and capsule signs may aid differential diagnosis between these two subtypes [6]. Chromophobe RCC, the third most common histologic subtype of RCC after CCRCC and PRCC, typically localizes in the periphery and presents as a well-defined and hypovascular mass, which is quite similar to Xp11.2 RCC and PRCC [24]. Further studies are required to address the differential diagnosis among these subtypes of RCC.

### 4.5. Limitations

There are some limitations with this study. Firstly, the sample size of Xp11.2 RCC and PRCC was relatively small [25]. Secondly, the role of other imaging methods such as MRI, positron emission tomography (PET), and more innovative imaging techniques were not referred in this study [26, 27]. For example, PRCC frequently shows a pseudocapsule and has low signal intensity on both T1- and T2-weighted MRI [19], while Xp11.2 RCC often shows hyper- or isointense on T1-weighted image and heterogeneous intensity on T2-weighted image [8].

## 5. Conclusion

In conclusion, Xp11.2 RCC often affects young women with gross hematuria, while PRCC affects older men without specific symptoms. Most Xp11.2 RCCs involve both the cortex and medulla simultaneously and have clear boundary, presenting as heterogeneous masses with necrotic or cystic components and intratumoral hemorrhage, circular calcification inside or around the tumor. After the injection of contrast media, Xp11.2 RCC shows moderately prolonged heterogeneous enhancement on the DCE-CT. PRCCs present as well-defined, homogeneous, and hypovascular masses on the DCE-CT. Type 1 PRCC is often smaller and more homogeneous than type 2. Type 1 PRCC has more distinct margins and less cystic components than type 2. Xp11.2 RCC is significantly larger and more heterogeneous than type 1 PRCC. Xp11.2 RCCs can be cystic and with circular calcification, while type 1 PRCCs are solid lesions without cystic degeneration or necrosis. Xp11.2 RCC enhances heterogeneously, while type 1 PRCC enhanced homogenously on DCE-CT. Xp11.2 RCC has clearer boundary and more common cystic components than those of type 2 PRCC. Differentiating Xp11.2 RCC with different subtypes of PRCC preoperatively will be beneficial for treatment planning.

## Figures and Tables

**Table 1:** The clinical, pathological details and tumor characteristics on CT in Xp11.2 RCC and PRCC (including type 1 and type 2).

	Xp11.2 RCC (*n* = 25)	PRCC (*n* = 39)	Type 1 (*n* = 15)	Type 2 (*n* = 24)	Xp11.2 versus PRCC	Xp11.2 versus type 1	Xp11.2 versus type 2	Type 1 versus type 2
Gender (male/female)	9/16	28/11	12/3	16/8	0.005^*∗*^	0.007^*∗*^	0.032^*∗*^	0.368
Age range (years)	19~51	32~78	35~78	32~73	—	—	—	—
Mean age (years)	30.7 ± 8.7	54.8 ± 11.1	55.9 ± 12.2	54.2 ± 10.5	<0.001^*∗*^	<0.001	<0.001^*∗*^	0.634
Gross hematuria	12/13	7/32	2/13	5/19	0.010^*∗*^	0.026^*∗*^	0.046^*∗*^	0.553
Location (left/right)	7/18	23/16	8/7	15/9	0.015^*∗*^	0.109	0.015^*∗*^	0.571
Location (cortical/cortical-medullar/medullar)	1/23/1	8/28/3	4/11/0	4/17/3	0.131	0.089	0.159	0.311
Diameter (cm)	5.4 ± 2.2	5.0 ± 2.4	3.6 ± 1.6	5.8 ± 2.5	0.426	0.009^*∗*^	0.592	0.002^*∗*^
Boundary (clear/unclear)	21/4	25/14	13/2	12/12	0.084	0.819	0.011^*∗*^	0.020^*∗*^
Shape (regular/irregular)	14/11	25/14	12/3	13/11	0.517	0.123	1.000	0.102
Attenuation (homo-/heterogeneous)	5/20	20/19	12/3	8/16	0.012^*∗*^	<0.001^*∗*^	0.291	0.005^*∗*^
Nature (solid/cystic-solid/cystic)	2/22/1	30/9/0	15/0/0	15/9/0	<0.001^*∗*^	<0.001^*∗*^	<0.001^*∗*^	0.007^*∗*^
Hemorrhage (with/without)	10/15	9/30	3/12	6/18	0.148	0.191	0.263	0.718
Circular calcification (with/without)	10/15	6/33	1/14	5/19	0.027^*∗*^	0.022^*∗*^	0.146	0.233
Fat (with/without)	0/25	0/39	0/15	0/24	1.000	1.000	1.000	1.000
Tumor thrombosis (with/without)	3/22	1/38	0/15	1/23	0.128	0.163	0.317	0.423
Lymph node metastasis (with/without)	3/22	4/35	0/15	4/20	0.827	0.163	0.641	0.095
Distant metastasis (with/without)	2/23	1/38	0/15	1/23	0.315	0.261	0.576	0.357
Treatment (OR/OP/LR/LP/RA)^a^	6/0/12/7/0	7/7/8/16/1	0/4/3/7/1	7/3/5/9/0	0.044^*∗*^	0.005^*∗*^	0.103	0.126
Stage (I/II/III/IV)	17/2/5/1	27/7/3/2	14/1/0/0	13/6/3/2	0.397	0.222	0.341	0.075
Median follow-up time (months)	31	32	35	30	—	—	—	—
Survival rate	88%	97.4%	100%	95.8%	0.128	0.163	0.317	0.423

Note: Xp11.2 RCC: renal cell carcinoma associated with Xp11.2 translocation/TFE3 gene fusions; PRCC: papillary renal cell carcinoma; ^a^OR: open radical nephrectomy; OP: open partial nephrectomy; LR: laparoscopic radical nephrectomy; LP: laparoscopic partial nephrectomy; RA: radiofrequency ablation. ^*∗*^ *P* < 0.05.

**Table 2:** Dynamic contrast enhanced CT attenuation (HU) of all subtypes of renal cell carcinoma.

	Xp11.2 RCC (*n* = 25)	PRCC (*n* = 37)	Type 1 PRCC (*n* = 12)	Type 2 PRCC (*n* = 25)	*P*
Unenhanced phase	44.8 ± 11.2	39.8 ± 6.6	38.9 ± 6.3	40.2 ± 6.8	0.032^*∗*^
Corticomedullary phase	81.3 ± 41.0	66.0 ± 31.9	56.0 ± 16.7	70.8 ± 36.4	0.114
Nephrographic phase	93.1 ± 40.0	73.1 ± 20.0	71.4 ± 17.4	73.8 ± 21.4	0.021^*∗*^
Delayed phase	80.3 ± 32.5	66.7 ± 25.6	56.8 ± 18.9	72.3 ± 35.7	0.126

Notes: Xp11.2 RCC: renal cell carcinoma associated with Xp11.2 translocation/TFE3 gene fusions; PRCC: papillary renal cell carcinoma; ^*∗*^ *P* < 0.05 (Xp11.2 RCC versus PRCC).

## Abbreviations

ANOVA: Analysis of variance
AUC: Area under ROC curve
CCRCC: Clear cell renal cell carcinoma
CT: Computed tomography
DCE-CT: Dynamic contrast-enhanced computed tomography
FISH: Fluorescence in situ hybridization
HU: Hounsfield unit
IHC: Immunohistochemical
MRI: Magnetic resonance imaging
PET: Positron emission tomography
PRCC: Papillary renal cell carcinoma
RCC: Renal cell carcinoma
ROC: Receiver operating characteristic
ROI: Region of interest
TFE3: Transcription factor E3
Xp11.2 RCC: Renal cell carcinoma associated with Xp11.2 translocation/TFE3 gene fusions.
